# LncRNA DANCR represses Doxorubicin-induced apoptosis through stabilizing MALAT1 expression in colorectal cancer cells

**DOI:** 10.1038/s41419-020-03318-8

**Published:** 2021-01-06

**Authors:** Minmin Xiong, Mengshi Wu, Dan Peng, Weijun Huang, Zehong Chen, Haoxian Ke, Zewen Chen, Wu Song, Yonghua Zhao, Andy P. Xiang, Xiaomin Zhong

**Affiliations:** 1 Key Laboratory for Stem Cells and Tissue Engineering, Ministry of Education, Center for Stem Cell Biology and Tissue Engineering, Zhongshan School of Medicine, Sun Yat-Sen University, 510080 Guangzhou, China; 2 Center of Gastrointestinal Surgery, Center of Gastric Cancer, The First Affiliated Hospital, Sun Yat-Sen University, 510080 Guangzhou, China; 3 School of Biological Science, University of Reading, Reading, RG6 6AH UK; 4 State Key Laboratory of Quality Research in Chinese Medicine, Institute of Chinese Medical Sciences, University of Macau, Taipa, Macao, 999078 Macao SAR, China

**Keywords:** Oncogenes, Long non-coding RNAs

## Abstract

Long non-coding RNA (lncRNA) DANCR has been reported to participate in key processes such as stem cell differentiation and tumorigenesis. In a high throughput screening for lncRNAs involved in Doxorubicin-induced apoptosis, we found DANCR was suppressed by Doxorubicin and it acted as an important repressor of apoptosis in colorectal cancer. Further studies demonstrated that DANCR promoted the oncogenic lncRNA MALAT1 expression via enhancing the RNA stability of MALAT1 to suppress apoptosis. MALAT1 could efficiently mediate the suppressive function of DANCR on apoptosis. Mechanistic studies found the RNA-binding protein QK served as an interacting partner of both DANCR and MALAT1, and the protein level of QK was subjected to the regulation by DANCR. Furthermore, QK was able to modulate the RNA stability of MALAT1, and the interaction between QK and MALAT1 was controlled by DANCR. In addition, QK could mediate the function of DANCR in regulating the expression of MALAT1 and suppressing apoptosis. These results revealed DANCR played a critical role in Doxorubicin-induced apoptosis in colorectal cancer cells, which was achieved by the interaction between DANCR and QK to enhance the expression of MALAT1.

## Introduction

Long non-coding RNA (lncRNA) is a category of RNA molecules arbitrarily defined as longer than 200 nucleotides, which usually has little protein-coding potential. Functions of lncRNAs have been widely studied in different biological systems, and they were found critical to many physiological processes, such as embryonic development and human diseases, especially cancers, by regulating cell proliferation, apoptosis, migration, metabolism, and differentiation^1–6^. Among the vast population of lncRNA species, a subset of lncRNAs have been reported to be involved in the control of cellular apoptosis. LncRNAs such as lincRNA-p21^7^, PANDA^8^, DINO^9^, PURPL^10^, NEAT1^11,12^, and PINCR^13^, are transcriptionally regulated by the tumor suppressor TP53 in response to DNA damage and apoptosis. In addition, lncRNAs H19, GAS5, TUG1, etc., were also found to be critical regulators of apoptosis in different cellular contexts^14–17^. Although these studies implicate the importance of lncRNAs in modulating apoptosis, the identities and biological functions of the majority of apoptosis-regulating lncRNAs remain to be elucidated under different physiological conditions, e.g. chemotherapeutic drugs-induced apoptosis in cancer cells.

Doxorubicin (Dox) is an anthracycline glycoside antibiotic that has been widely used as a chemotherapeutic drug for the treatment of a wide range of cancers for over 30 years^18^. Dox treatment generally elicits multiple physiological responses, such as apoptosis, autophagy, and necrosis. Apoptosis represents one of the typical phenotypes induced by Dox. The mechanism involved in Dox-induced apoptosis is especially important for resolving the problem of drug resistance to Dox that evolves in advanced tumors frequently^19^. Previous research indicated Dox activates AMP-activated protein kinase (AMPK-inducing apoptosis), TP53, and the Bcl-2/Bax apoptosis pathway to induce apoptosis^20–22^. Recently, studies found lncRNAs were emerging as novel regulators in Dox-induced apoptosis. Chang group identified lncRNA DINO served as a TP53-controlled transcript upon Dox treatment. DINO was necessary for TP53-dependent cell cycle arrest and apoptosis by binding to and stabilizing the TP53 protein^9^. Similarly, Lal group found lncRNA PURPL formed a regulatory loop with TP53 in Dox-induced apoptosis and modulated tumorigenesis in colorectal cancer cells^10^.

To get a full insight of lncRNAs involved in the mechanism controlling Dox-induced apoptosis, this study took advantage of the RNA sequencing data from Lal group^10^ to identify novel lncRNA regulators. We found the expression of differentiation antagonizing non-protein coding RNA (DANCR) was suppressed upon Dox treatment, implicating it might be involved in Dox-induced apoptosis in colorectal cancer cells. LncRNA DANCR was initially identified as a suppressor of epidermal progenitor cell differentiation^3^. Studies indicated DANCR could affect osteoblast differentiation through association with enhancer of zeste homolog 2 (EZH2) to repress the expression of RUNX2 gene^23^. In addition, DANCR was reported to overexpress in multiple types of cancers, implicating its important function in tumorigenesis^24–30^. For example, in prostate cancer DANCR was identified as a downstream gene of C-MYC, and DANCR promoted cell cycle progression and cell proliferation by repressing the expression of CDKN1A^24^. In hepatocellular cancer, DANCR elevated the expression of CTNNB1 to maintain the cancer stem cell population and induced the development of xenograft liver cancer in mouse model^29^. And DANCR was reported to interact with the HIF1α pathway to enhance the metastasis of nasopharyngeal carcinoma^30^. In addition, DANCR had also been demonstrated to be involved in the proliferation, metastasis, angiogenesis, and differentiation in various cancer types, such as osteosarcoma, glioma, breast cancer, cervical cancer, bladder cancer, etc.^25–28,31–33^. Based on these studies, DANCR is emerging as an oncogenic lncRNA in cancers.

However, the contribution of DANCR to the tumorigenesis of colorectal cancer and its working mechanism have not been fully elucidated. So far only a few studies have explored the function of DANCR in colorectal cancer. A meta-analysis report based on a Chinese population showed that a high expression level of DANCR was correlated to the progression and poor prognosis of colorectal cancer^34^. Zeng group reported that the overexpression of DANCR in colorectal cancer was correlated with cell proliferation and cancer metastasis^35^. Tao et al. found that down regulation of DANCR induced cell death and the expression of apoptotic markers^36^. Although these studies provided early evidence depicting the oncogenic role of DANCR in colorectal cancer, the major function and detailed working mechanism of DANCR in the disease need further exploration.

In this work, we reported lncRNA DANCR repressed by Dox was closely correlated with colorectal cancer cell apoptosis. The suppressive function of DANCR on apoptosis was attributed to regulating the expression of MALAT1. Furthermore, the interaction between DANCR and the RNA-binding protein QK contributed to the regulatory function of DANCR on MALAT1 expression and suppression of apoptosis. In summary, these findings provide new insight into the function and the working mechanism of DANCR in colorectal cancer cells.

## Results

### DANCR was overexpressed in colorectal cancer with a regulatory function in cell growth

**Fig. 1:**
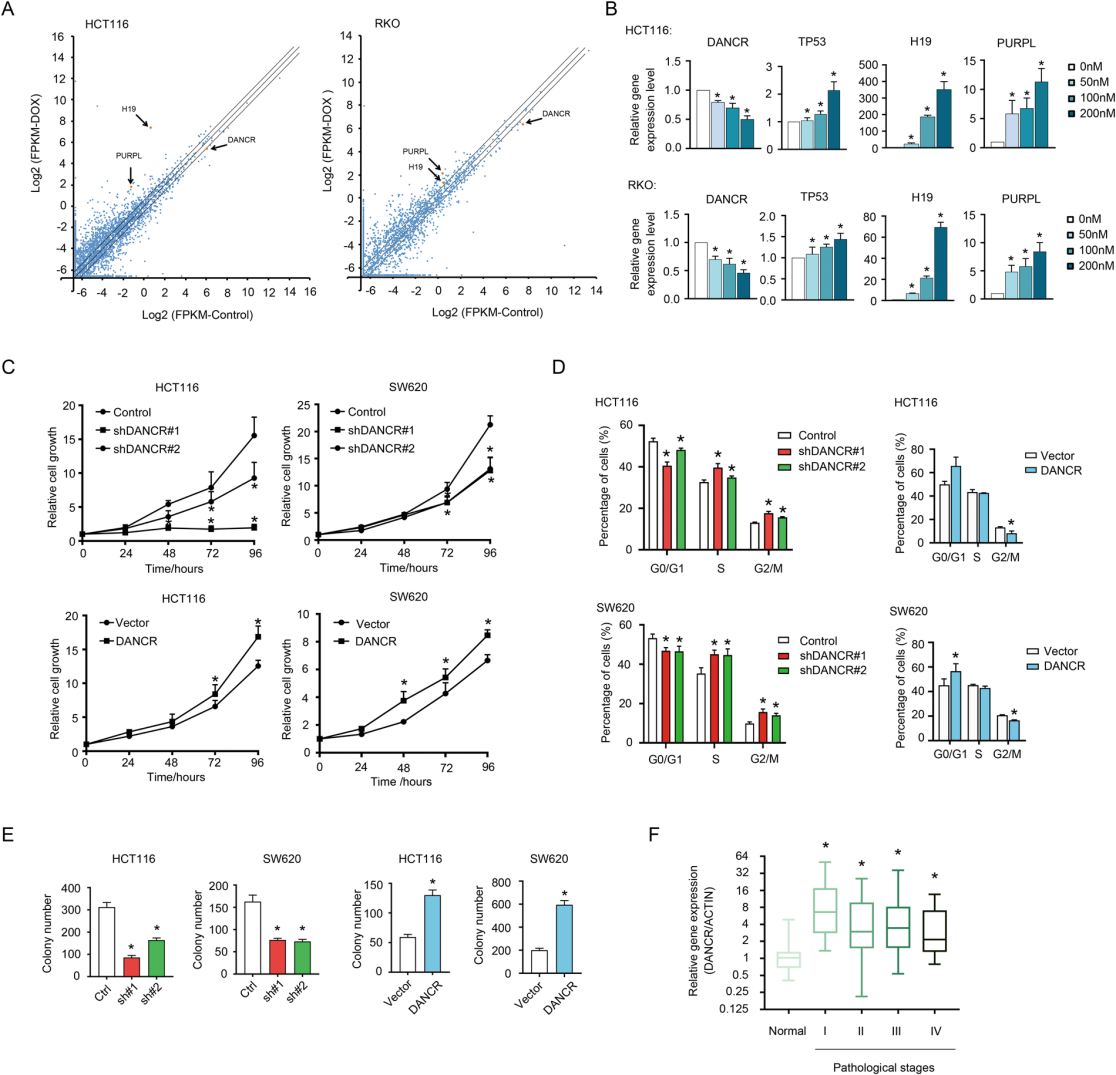
DANCR was closely correlated with cell growth and tumor progression in colorectal cancer. **A** Changes in lncRNA expression in HCT116 and RKO cells upon Dox treatment were presented in Scatter Dot Plots. *X* axis: log_2_(FPKM) of control cells; *Y* axis: log_2_(FPKM) of Dox-treated cells. Arrows indicated lncRNAs DANCR, PURPL, and H19, whose fold changes were higher than 1.5-fold. **B** The expression levels of DANCR, TP53, H19, and PURPL were detected by qPCR in HCT116 (top) and RKO (bottom) cells treated with increasing dosages of Dox (0, 50, 100, 200 nM) for 24 h. *p < 0.05. **C** DANCR knockdown inhibited HCT116 and SW620 cells proliferation (top), whereas DANCR overexpression promoted the growth of HCT116 and SW620 cells detected by CCK8 assays (bottom). *p < 0.05. **D** Cell cycle progression was analyzed using flow cytometry in HCT116 and SW620 cells with shDANCR or DANCR overexpression vectors. *p < 0.05. **E** The anchorage-independent growth capacity of HCT116 and SW620 cells was impaired upon DANCR knockdown, and it was enhanced in HCT116 and SW620 cells overexpressing DANCR. *p < 0.05. **F** Relative expression levels of DANCR in colorectal cancer tissues of different pathological stages. Normal, adjacent noncancerous tissues (*n* = 18). I–IV: TNM stages I (*n* = 21), II (*n* = 40), III (*n* = 44), and IV (*n* = 21). *p < 0.05.

To identify novel lncRNAs involved in Dox-induced apoptosis, data from the GEO dataset GSE79249 was retrieved and analyzed to detect changes in gene expression upon Dox treatment in wild type HCT116 cells (GSM2089687/GSM2089688: Control vs. GSM2089689/GSM2089690: Dox), and in wild type RKO cells (GSM2089693: Control vs. GSM2089694: Dox)^10^. LncRNA genes with a cutoff of 1.5-fold change were sorted out for further investigation. Results of HCT116 and RKO cells were compared. The two cell lines had 449 upregulated and 154 downregulated lncRNAs in common (Fig. 1A, Table S2). LncRNAs previously demonstrated to be correlated with apoptosis regulation, such as PURPL and H19^10,14,37,38^, were identified upregulated upon Dox treatment (Fig. 1A). And DANCR turned out to be one of the downregulated genes in both HCT116 and RKO cells (Fig. 1A), which could be independently verified by treating the two cell lines with increasing dosages of Dox (Fig. 1B). As controls, the expression levels of TP53, H19, and PURPL were also validated to be positively correlated to Dox dosage. These results implicated a close relationship of DANCR with Dox-induced apoptosis. To explore the role of DANCR in cell growth and apoptosis, we constructed the loss-of-function and gain-of-function stable cell lines with both HCT116 and SW620 cells (Fig. S1A, S1B). When DANCR was silenced with two independent shRNAs, both HCT116 and SW620 cells first showed retarded proliferation (Fig. 1C, top), and subsequently severe cell death. And overexpression of DANCR promoted the growth of both HCT116 and SW620 cells moderately (Fig. 1C, bottom). Furthermore, DANCR knockdown resulted in decreased cell numbers in G0/G1 phase but elevated cell numbers in S and G2/M phases (Fig. 1D, left; Fig. S1C), and overexpression of DANCR had an opposite effect in both HCT116 and SW620 cells (Fig. 1D, right; Fig. S1C). In addition, colony forming assays indicated DANCR significantly promoted the capacity in anchorage-independent growth of HCT116 and SW620 cells (Figs. 1E and S1D). However, DANCR had minor effects on the migration activity of HCT116 and SW620 cells (Fig. S1E, S1F). To further evaluate the contribution of DANCR to colorectal cancer progression, an expression profiling for DANCR was performed with commercially available cDNA arrays derived from human colon cancer tissues (cancerous tissues of TNM Stages I–IV, *n* = 126; adjacent normal tissues, *n* = 18). DANCR expression was significantly higher in malignant tissues, especially in Stage I, than in normal tissues (Fig. 1F). However, no significant correlations between DANCR expression and regional lymph node metastasis, neither distant metastasis, were observed (data not shown). These results indicated the high expression level of DANCR might contribute to the early development of colorectal cancer.

### DANCR-regulated apoptosis of colorectal cancer cells in vitro

**Fig. 2:**
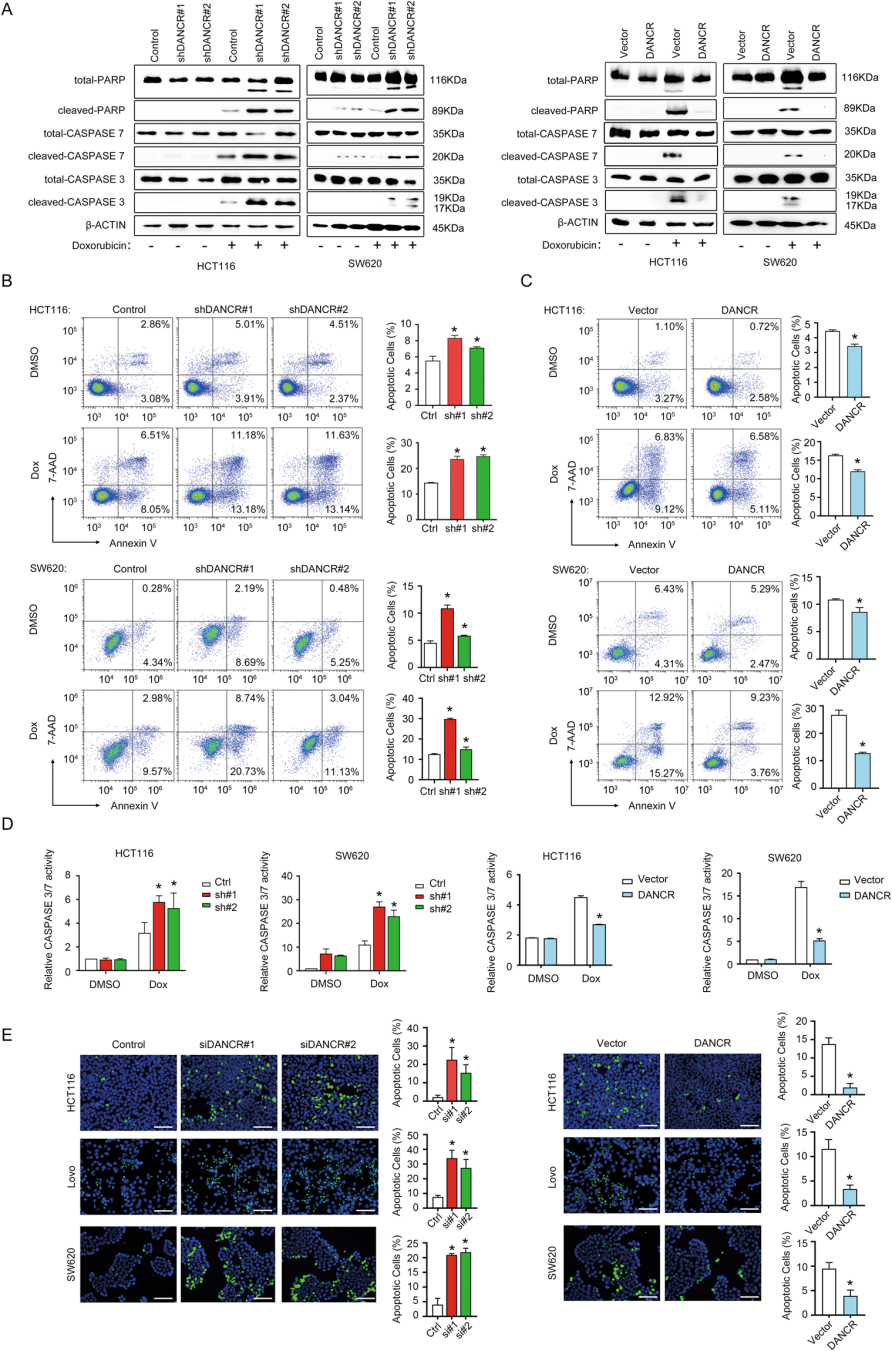
DANCR regulated apoptosis of colorectal cancer cells in vitro. **A** Left: Western blotting showed increased expression of total and cleaved PARP, CASPASE 7, and CASPASE 3 upon treatment with 200 nM Doxorubicin for 24 h in HCT116 and SW620 cells with shDANCR. Right: DANCR overexpression in HCT116 and SW620 cells repressed the expression of cleaved PARP, cleaved CASPASE 7, and cleaved CASPASE 3 detected by Western blotting. **B** Representative images of Annexin V flow cytometry assays and data statistics showed increased apoptotic cell numbers upon DANCR silencing in HCT116 and SW620 cells, which was treated with DMSO or 200 nM Doxorubicin for 24 h. *p < 0.05. **C** Annexin V flow cytometry assays showed DANCR overexpression increased tolerance to apoptosis in HCT116 and SW620 cells treated with DMSO or 200 nM Doxorubicin for 24 h. *p < 0.05. **D** The effects of DANCR depletion and DANCR overexpression on the CASPASE 3/7 activity in HCT116 and SW620 cells in the presence or absence of 200 nM Doxorubicin for 24 h. *p < 0.05. **E** TUNEL staining and statistical analysis in HCT116, LoVo, and SW620 cells with DANCR siRNAs (left) or with DANCR overexpressing vectors (right). Bar, 100 μm. *p < 0.05.

As mentioned above, severe cell death was observed following growth retardation upon loss-of-function of DANCR. We hypothesized cell death represented the major phenotype resulted from DANCR knockdown. To verify the hypothesis, protein markers of apoptosis (PARP, CASPASE 7, and CASPASE 3) were examined when HCT116 and SW620 cells were treated with Dox (Fig. 2A). We found the cleaved forms of PARP, CASPASE 7, and CASPASE 3 were all elevated in shDANCR cell lines of HCT116, SW620, and HT-29 compared to control cells in the presence of Dox (Fig. 2A, left; Fig. S2A). On the other hand, overexpression of DANCR in HCT116 and SW620 cells decreased the expression of cleaved PARP, CASPASE 7, and CASPASE 3 (Fig. 2A, right). Annexin V flow cytometry assay further confirmed DANCR knockdown significantly increased (Figs. 2B and S2B), and overexpression of DANCR decreased (Fig. 2C), the number of apoptotic cells in HCT116, SW620, and HT-29 cell lines. An in vitro assay to measure the enzymatic activity of CASPASE 3/7 from cell lysates displayed that DANCR knockdown increased, while DANCR overexpression decreased, the enzymatic activity in the presence of Dox in both HCT116 and SW620 cells (Fig. 2D). In addition, TUNEL assay showed that HCT116, LoVo, and SW620 cells with DANCR siRNAs had a higher number of apoptotic cells than control cells (Fig. 2E, left). Nevertheless, DANCR overexpression had an opposite effect (Fig. 2E, right). From the above data, we concluded that repression of apoptosis was a major biological role of DANCR in colorectal cancer cells in vitro.

### DANCR suppressed apoptosis of colorectal cancer cells in vivo

**Fig. 3:**
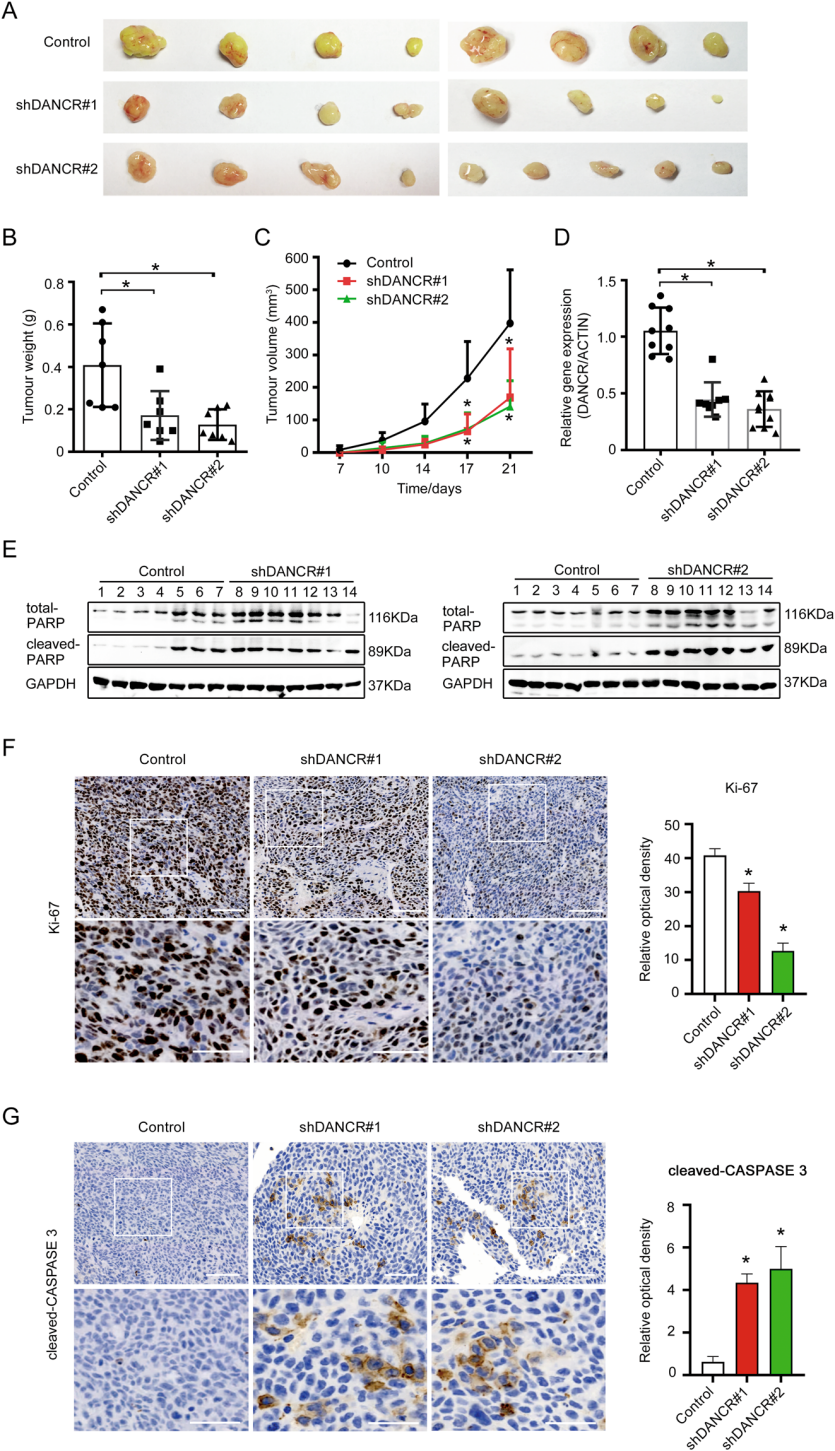
DANCR suppressed apoptosis of colorectal cancer cells in vivo. **A** Morphology of xenograft tumors formed by HCT116 cells with DANCR shRNAs or control cells. **B**, **C** Comparison of the weight and the size of xenograft tumors as shown in **A**. *p < 0.05. **D** The expression of DANCR in xenograft tumors as shown in **A** was analyzed by qRT-PCR. *p < 0.05. **E** The expression of cleaved PARP in xenograft tumors was detected by Western blotting. **F**, **G** The expression of Ki-67 and cleaved CASPASE 3 in xenograft tumors was showed in representative immunohistochemical images (left). Relative optical density of the expression of Ki-67 and cleaved CASPASE 3 was analyzed (right). Bar, 100 μm (upper panel) and 50 μm (lower panel). *p < 0.05.

To verify whether DANCR suppresses apoptosis in vivo to promote tumor growth, we performed xenograft tumor formation assays by implanting HCT116 shDANCR cells or control cells subcutaneously to nude mice. Tumor samples harvested 3 weeks post-implantation indicated shDANCR cells formed tumors with dramatic decrease in the average size (Fig. 3A), as well as decreases in tumor weights (Fig. 3B) and tumor volumes (Fig. 3C). The silencing efficiency of DANCR expression was validated in the xenograft tumors (Fig. 3D), indicating the difference in tumor size was specifically correlated with DANCR expression level. To ensure cellular apoptosis was responsible for the negative effect that DANCR exerted to tumor formation in vivo, the expression of PARP, Ki-67, and CASPAPSE 3 was examined in the xenograft tumor samples. Results showed that cleaved PARP expressed at a higher level in shDANCR groups than in the control group (Fig. 3E). Immunohistochemistry (IHC) also demonstrated that shDANCR groups had significantly fewer Ki-67-positive cells, and much more cleaved CASPASE 3-positive cells than the control group (Fig. 3F, G). These data suggested that DANCR enhanced tumor formation in vivo through regulating apoptosis.

### DANCR modulated MALAT1 expression by enhancing the RNA stability of MALAT1

**Fig. 4:**
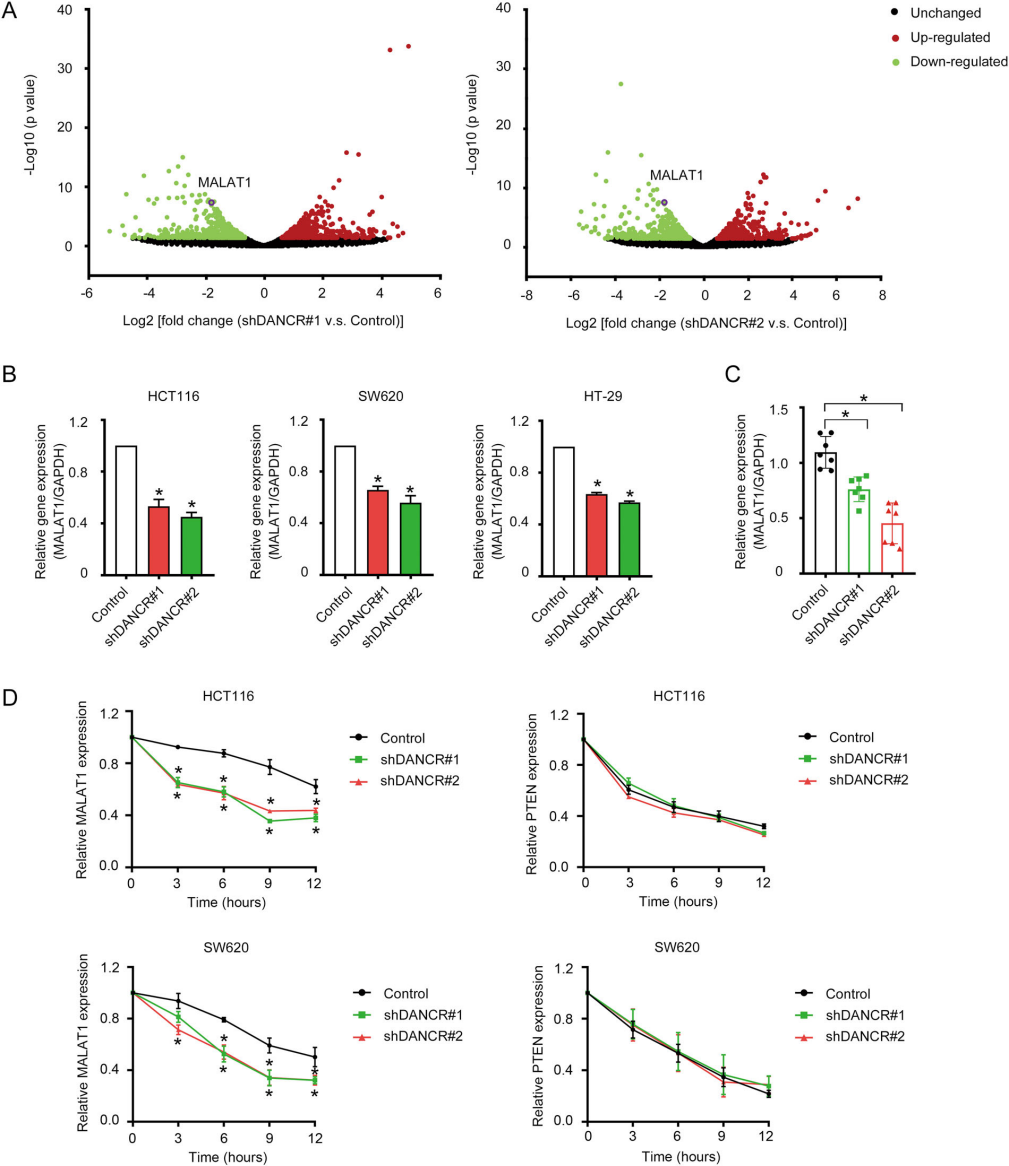
DANCR modulated MALAT1 expression by enhancing the RNA stability of MALAT1. **A** Differentially expressed genes upon DANCR knockdown in HCT116 cells are shown in volcano plots. *X* axis: Log_2_ [fold change (shRNA vs. Control)]; *Y* axis: −Log_10_ (*p* value). **B** Decreased expression of MALAT1 upon DANCR knockdown was validated by qRT-PCR in HCT116, SW620, and HT-29 cells. *p < 0.05. **C** Decreased expression of MALAT1 upon DANCR knockdown was validated using qRT-PCR in xenograft tumor tissues (*n* = 7 for each group). *p < 0.05. **D** The expression levels of *MALAT1* (left) and *PTEN* (right) upon DANCR knockdown in HCT116 and SW620 cells were detected by qRT-PCR at 0, 3, 6, 9, and 12 h post-treatment with Actinomycin D. *p < 0.05.

In order to investigate the anti-apoptotic mechanism of DANCR and to get a genome-wide insight of the molecular changes induced by DANCR knockdown, we performed a transcriptome sequencing using the shDANCR and control HCT116 cell lines. DANCR knockdown led to changes in the expression of a large number of genes (Fig. 4A and Table S3), including some genes known to be correlated with cell proliferation and apoptosis, such as MALAT1, XIAP, SOCS2, CCND1, etc. Among the altered genes, we were particularly interested in MALAT1, whose expression was significantly downregulated upon DANCR knockdown (Fig. 4A), due to its widely studied functions in cancers, including promotion of cell growth and metastasis, and suppression of apoptosis^39–45^. As shown in Fig. 4B, the regulation to MALAT1 expression by DANCR could be consistently validated in HCT116, SW620, and HT-29 cell lines. Furthermore, in xenograft tumor tissues (as shown in Fig. 3), DANCR knockdown also dramatically impaired MALAT1 expression (Fig. 4C).

To explore whether DANCR modulated MALAT1 expression transcriptionally, reporter assays using a *MALAT1* promoter-driven Luciferase plasmid were performed in shDANCR HCT116 cells. However, DANCR knockdown only had minor effects on the transcriptional activity of *MALAT1* promoter (Fig. S3). Subsequently, the RNA stability of MALAT1 was examined in HCT116 and SW620 cell lines with shDANCR vectors (Fig. 4D, left). Surprisingly, MALAT1 in shDANCR cells degraded much faster than in control cells. The difference in the turn-over rate of MALAT1 transcript could already be seen as early as 3 h post-treatment of Actinomycin D. However, an irrelevant mRNA *PTEN* did not manifest differences in the degradation dynamics between shDANCR cells and control cells (Fig. 4D, right), indicating DANCR regulated the RNA stability of MALAT1 specifically.

### MALAT1 mediated the anti-apoptotic function of DANCR

**Fig. 5:**
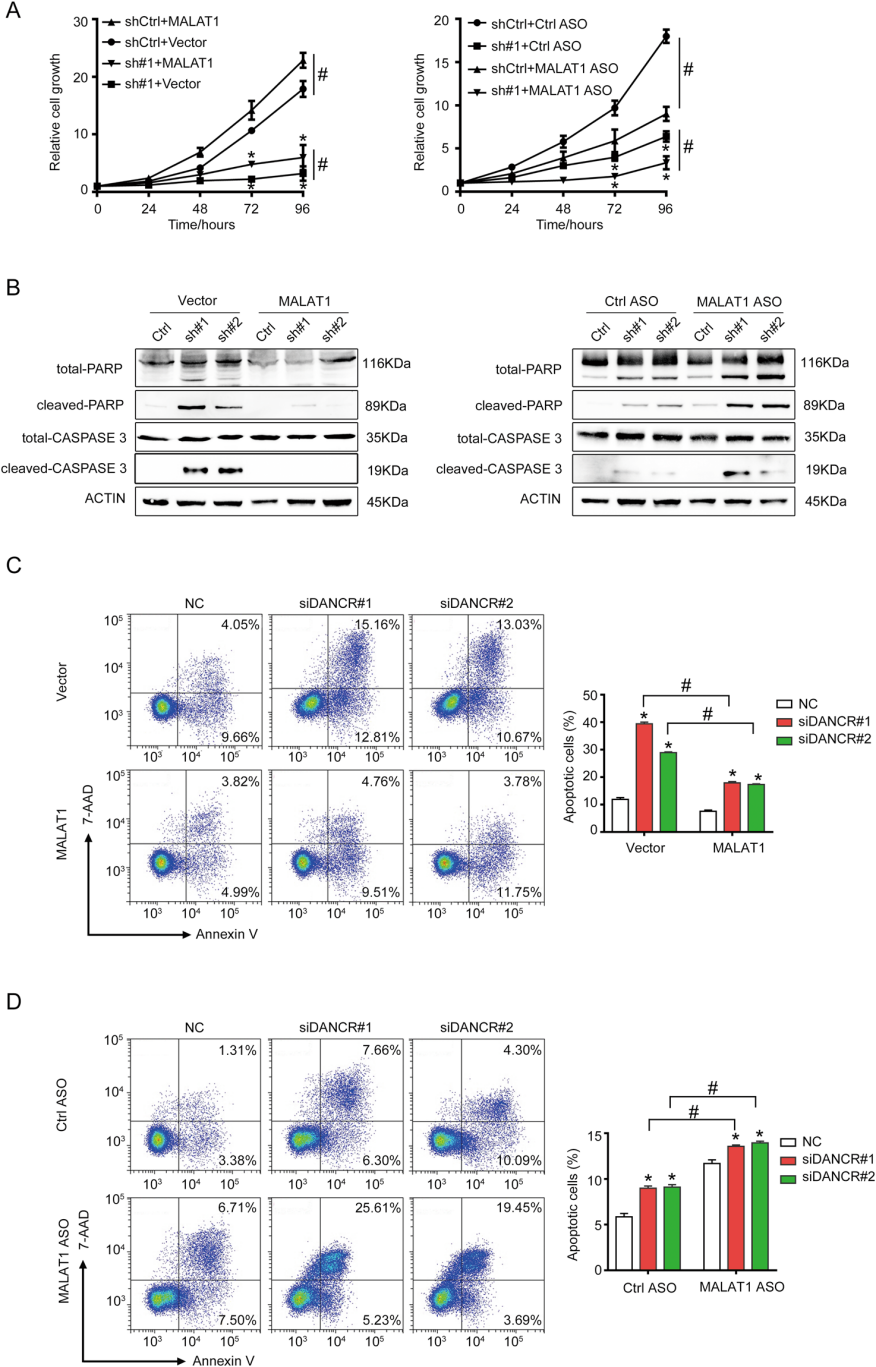
MALAT1 mediated the anti-apoptotic function of DANCR. **A** MALAT1 overexpression could partially rescue the growth inhibition caused by DANCR silencing (left), and simultaneously silencing MALAT1 in shDANCR HCT116 cells further inhibited cellular proliferation (right). sh#1, DANCR shRNA#1. ASO antisense oligonucleotides. * and #, *p* < 0.05. **B** Western blotting detected MALAT1 overexpression decreased (left), while MALAT1 depletion further increased (right), the expression of cleaved PARP and cleaved CASPASE 3 induced by DANCR silencing. **C** MALAT1 overexpression decreased DANCR knockdown-induced apoptosis detected with Annexin V flow cytometry assay. Representative images and statistical analysis were shown. * and # *p* < 0.05. **D** MALAT1 depletion further increased DANCR knockdown-induced apoptosis detected with Annexin V flow cytometry assay. Representative images and statistical analysis were shown. * and #, *p* < 0.05.

As shown above, DANCR regulated the RNA stability of MALAT1. To prove whether the regulation to MALAT1 expression by DANCR had physiological functions, we tested the possibility of MALAT1 to mediate the anti-apoptotic function of DANCR. MALAT1 was overexpressed or knockdown by antisense oligonucleotides (ASO) in HCT116 shDANCR cells (Fig. S4). Cell proliferation assays showed that overexpression of MALAT1 rescued the growth retardation induced by DANCR knockdown (Fig. 5A, left). And simultaneously silencing MALAT1 further compromised the growth of shDANCR cells (Fig. 5A, right). Meanwhile, the expression of cleaved PARP and cleaved CASPASE 3 proteins in shDANCR cells overexpressing MALAT1 was decreased to an almost undetectable level compared to parental shDANCR cells (Fig. 5B, left). However, silencing MALAT1 in shDANCR cells dramatically promoted the production of cleaved PARP and cleaved CASPASE 3 proteins (Fig. 5B, right). Similarly, Annexin V assays showed that the population of apoptotic cells induced by DANCR knockdown was significantly decreased when simultaneously overexpressing MALAT1 (Fig. 5C). And silencing MALAT1 in siDANCR cells further enhanced the number of apoptotic cells compared to parental siDANCR cells (Fig. 5D). The above data implicated that MALAT1 could mediate the suppressive function of DANCR in apoptosis.

### DANCR-associated protein QK promoted the stability of MALAT1

**Fig. 6:**
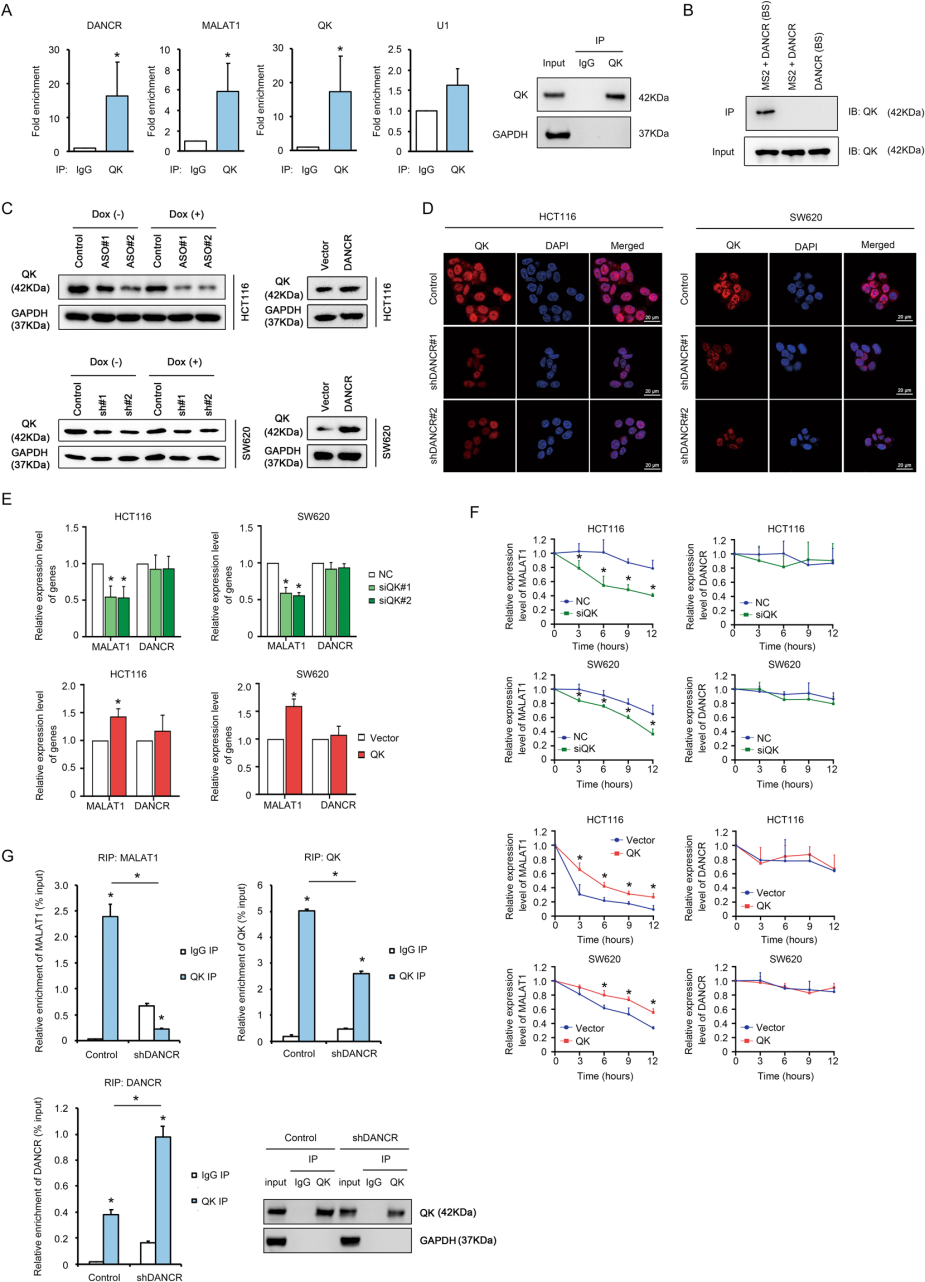
DANCR-associated protein QK promoted the stability of MALAT1. **A** Left: Fold enrichment of DANCR, MALAT1, QK mRNA, and U1 from the RIP assay with a QK antibody in HCT116 cells was detected by qPCR. Right: Immunoprecipitation of QK protein from the RIP assay was shown by Western Blotting. **B** The enrichment of QK protein from the MS2-DANCR RIP assay in HCT116 cells was detected by Western Blotting. MS2, the MS2 RNA-binding protein. DANCR (BS), ectopic expressed DANCR with a tandem sequence of 12-copied MS2-binding site. **C** The changes in QK protein level upon DANCR knockdown (left) or overexpression (right) in HCT116 and SW620 cells was detected by Western Blotting in the presence or absence of 200 nM Dox for 24 h. ASO, antisense oligonucleotide of DANCR. sh#1/#2, shDANCR#1/#2. **D** Immunofluorescence staining showed the expression of QK protein in HCT116 and SW620 cells with control vector, shDANCR#1, and shDANCR#2. Red: QK, Blue: DAPI. Bar, 20 μm. **E** The expression levels of MALAT1 and DANCR in siQK (top) and QK overexpression cells (bottom) of HCT116 and SW620 were detected by qPCR. *p < 0.05. **F** The dynamic expression changes of MALAT1 and DANCR in siQK and QK overexpression cells of HCT116 and SW620 were detected by qRT-PCR at 0, 3, 6, 9, and 12 h post-treatment with Actinomycin D. *p < 0.05. **G** The relative enrichment of immunoprecipitated RNAs (% input) from control and shDANCR HCT116 cell lines were compared by qPCR. QK protein in the cell lysate input and in the immunoprecipitate was detected by Western Blotting. *p < 0.05.

Although DANCR was confirmed to repress apoptosis by enhancing the expression of MALAT1, the question how DANCR modulated MALAT1 stability remained unanswered. We hypothesized the regulation to MALAT1 expression by DANCR was implemented by direct RNA–RNA interaction. However, the results of RNA in situ hybridization in wide type HT29 and HCT116 cell lines did not support this hypothesis. Only a negligible portion of DANCR, whose subcellular localization was detected 80% in the cytoplasm and 20% in the nucleus, overlapped with MALAT1 inside the nucleus (Fig. S5). Considering the evidence that lncRNAs usually associate with proteins to perform their functions, we searched *Starbase* (http://starbase.sysu.edu.cn)^46^ for RNA-binding proteins that may interact with DANCR. Interestingly, we found DANCR, as well as MALAT1, harbored multiple binding sites of the RNA-binding protein QUAKING (QK), which is a widely reported regulator to RNA stability, RNA splicing, RNA transportation, and translational efficiency^47–50^. Furthermore, QK has been previously reported to be involved in apoptosis regulation^51^. Therefore, we hypothesized that QK served as an interacting partner of DANCR to mediate the regulatory function of DANCR in MALAT1 expression. First, we sought to confirm the interaction between DANCR and QK. The RNA-binding protein immunoprecipitation (RIP) assay with a QK specific antibody showed significant enrichment of DANCR and MALAT1 by QK (Fig. 6A). As controls, QK mRNA and U1, respectively, displayed abundant and negligible enrichment compared to control IgG IP. This indicated the interaction between DANCR and QK, as well as MALAT1 and QK, was highly specific. Subsequently, an MS2-DANCR RIP assay was developed to validate the above result using the strategy that DANCR transcript expressed with a tandem sequence of 12-copied MS2 protein-binding sites (DANCR (BS)), together with the interacting partners of DANCR, can be specifically enriched by the MS2 protein^52^. Results showed that QK protein was efficiently captured in the presence of both MS2 protein and DANCR (BS), but not in the negative control with MS2 protein and wild type DANCR, nor the one with DANCR (BS) alone without MS2 protein (Fig. 6B). Interestingly, QK mRNA and MALAT1 were not significantly enriched in the MS2+ DANCR (BS) immunoprecipitate (Fig. S6). This implicated QK protein, instead of its mRNA, was the interacting partner of DANCR, and MALAT1 might not have direct interaction with DANCR. To determine the physiological role of the interaction between QK protein and DANCR, we tested whether DANCR could affect the expression of QK. When DANCR was knocked down by either ASO or shRNA, the protein level of QK detected by Western Blotting (Fig. 6C, left) and immunofluorescence staining (Fig. 6D) was significantly diminished in both HCT116 and SW620 cells. And DANCR overexpression elevated the protein level of QK moderately (Fig. 6C, right). This result indicated DANCR interacted with QK to play a key role in maintaining the protein level of QK.

Since MALAT1 could also be enriched by QK (Fig. 6A), we next verified whether QK was responsible for maintaining the steady-state expression level and the RNA stability of MALAT1. QK knockdown by siRNA remarkably decreased MALAT1 expression level, and QK overexpression increased MALAT1 level (Figs. 6E and S7). However, the expression level of DANCR was unaltered by QK. Subsequently, the effect of QK on the RNA stability of MALAT1 was further investigated by treating the QK knockdown and overexpression cell lines with Actinomycin D. In accordance with the result of Fig. 6E, QK knockdown dramatically accelerated the turnover rate of MALAT1, and QK overexpression significantly enhanced the RNA stability of MALAT1 in HCT116 and SW620 cells (Fig. 6F, left). And under the same conditions, QK did not affect the turnover of DANCR (Fig. 6F, right). Therefore, we concluded QK participated in the maintenance of MALAT1 expression level. To further characterize the mechanism through which QK regulated MALAT1 expression, RIP assays with a QK-specific antibody was performed in HCT116 cells with shDANCR (Figs. 6G and S8). In control cells, QK protein could efficiently enrich DANCR, MALAT1, and QK mRNA compared to control IgG IP. Upon DANCR knockdown by shRNA, the interaction between QK and MALAT1 was abrogated to a large extent, which was comparable to the enrichment of MALAT1 in control IgG IP group (Fig. 6G, top left). Nevertheless, the enrichment of QK mRNA only decreased by about 50% in shDANCR cells (Fig. 6G, top right), which was proportional to the decrease in QK protein upon DANCR knockdown (Fig. 6G, bottom right). Consequently, we speculated the dramatic reduction in the interaction between QK and MALAT1 upon DANCR knockdown was probably due to the control by DANCR, more than due to the decrease in the input QK protein. These results implicated DANCR was a master regulator to the association between QK and MALAT1, and hence the RNA stability of MALAT1 and the resistance to apoptosis.

### QK mediated the function of DANCR on regulating MALAT1 expression and suppressing apoptosis

**Fig. 7:**
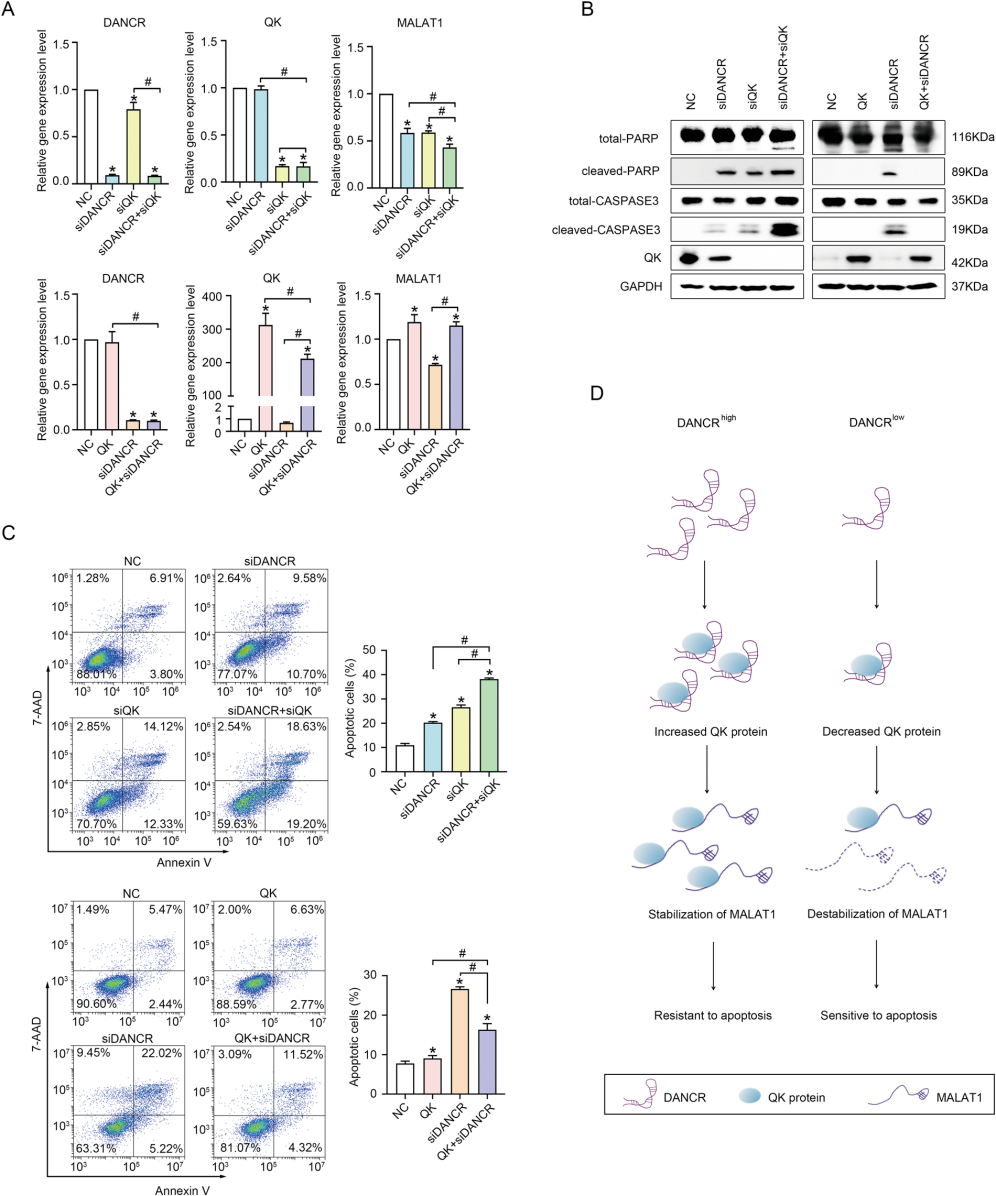
QK mediated the function of DANCR on regulating MALAT1 expression and suppressing apoptosis. **A** The expression of MALAT1 upon DANCR knockdown combined with siQK (upper panel), or with QK overexpression (lower panel) respectively, was detected by qRT-PCR in HCT116 cells. NC, negative control. *and #, *p* < 0.05. **B** Western blotting detected QK depletion further increased (left panel), while QK overexpression inhibited (right panel), the expression of cleaved PARP and cleaved CASPASE 3 induced by DANCR silencing in HCT116 cells. **C** QK depletion (upper panel) and QK overexpression (lower panel) further aggravated or alleviated siDANCR-induced apoptosis, which was detected with Annexin V flow cytometry assay in HCT116 cells. Representative images and statistical analysis were shown. *and #, *p* < 0.05. **D** A schematic diagram for the mechanism of apoptosis regulation by the DANCR/QK/MALAT1 axis in colorectal cancer cells.

To further characterize the functional correlation between the regulation to QK protein by DANCR and the stabilization of MALAT1 by QK protein, we tested whether QK could cooperate with DANCR in modulating MALAT1 expression and suppressing apoptosis. In HCT116 cells with siDANCR, simultaneously silencing QK further decreased MALAT1 expression to a level much lower than in cells with siDANCR or siQK alone (Fig. 7A, top right), and co-expressing a QK cDNA efficiently rescued the downregulated expression of MALAT1 to a level comparable to control cells (Fig. 7A, bottom right). The expression of apoptotic marker proteins demonstrated that siQK further increased cleaved PARP and cleaved CASPASE 3 expression induced by siDANCR, and QK overexpression in siDANCR cells abrogated the production of cleaved PARP and cleaved CASPASE 3 (Fig. 7B). Annexin V assays also showed that co-silencing QK significantly elevated the apoptotic cell number induced by siDANCR (Fig. 7C, top), and QK overexpression in siDANCR cells reduced the apoptotic cell number to a level between the negative control and siDANCR alone (Fig. 7C, bottom). Summarized from the above data, we concluded that DANCR was a critical regulator to the protein level of QK, and QK served as a mediator for DANCR to perform its function on stabilizing MALAT1 expression and suppressing apoptosis (Fig. 7D).

## Discussion

Dox is a widely used chemotherapeutic drug for treating various types of cancers, and it triggers complicated physiological responses in different cellular contexts. The downstream pathways and targets regulated by Dox need further elucidation to overcome the problem of Dox resistance in advanced tumors. In this work, we revealed DANCR was a Dox-suppressed target, which was involved in the regulation of apoptosis in colorectal cancer (Figs. 2 and 3). Nevertheless, how Dox controlled DANCR expression, as well as whether DANCR had functional correlation with the previously known regulatory genes for apoptosis (such as TP53), remained questions unanswered. Hopefully, this data added DANCR as a new effector in the downstream pathway of Dox, which may help resolve the problem of drug resistance to Dox in multiple cancer types.

Our work indicated that the major function of DANCR in colorectal cancer resided in the regulation of apoptosis. Interestingly, transcriptome-wide analysis revealed DANCR was able to upregulate expression of MALAT1. MALAT1 has been proven a potent repressor for cancer cell apoptosis^41,45,53,54^. Further studies revealed MALAT1 could functionally mediate the suppressive function of DANCR on apoptosis. However, the mechanism through which MALAT1 repressed apoptosis in colorectal cancer remains unclear. In multiple myeloma, MALAT1 was shown to repress apoptosis through binding to components of the DNA damage repair complex to enhance alternative non-homologous end joining^42^, or controlling the expression of proteasome subunits and anti-oxidation genes^39^. In our study, whether MALAT1 mediated the anti-apoptotic function of DANCR through the same mechanism remains an interesting question for further investigation.

Our work also demonstrated DANCR suppressed apoptosis through enhancing the RNA stability of MALAT1. MALAT1 had been reported to form a stable RNA conformation through processing the 3′ end into a triple helix^55–58^. The RNA stability of MALAT1 was regulated by factors that can promote 3′ end processing and maturation, such as a natural antisense RNA TALAM1^57^ and the Microprocessor Drosha–DGCR8 complex^59^. In this work, we identified the RNA-binding protein QK as a mediator in transducing the positive regulation of DANCR to MALAT1. Although we identified QK could bind to both DANCR and MALAT1, it remains an interesting question how QK promotes the RNA stability of MALAT1. Whether QK participates in the 3′ end processing of MALAT1, or alternatively QK interacts with TALAM1 and Drosha–DGCR8 complex, need further investigation.

In addition, DANCR controlled the protein level of QK, as well as the interaction between QK and MALAT1. However, the majority of DANCR localizes in the cytoplasm (Fig. S5), and QK protein predominantly localizes inside the nucleus (Fig. 6D). It constitutes another interesting question how DANCR associates with QK and affects QK protein level. We speculated that cytoplasmic DANCR might regulate the protein stability of QK, or otherwise affect the transportation of QK from cytoplasm to nucleus, through direct interaction with QK protein. It is less likely that DANCR binds to and stabilizes QK mRNA to promote the translation of QK protein, because QK mRNA showed minor enrichment in MS2-DANCR RIP assays (Fig. S6). And the expression level of QK mRNA even elevated moderately upon DANCR knockdown (Fig. S8), which was opposite to the changes in QK protein level, probably due to a negative feedback from the decrease in QK protein (Fig. 6G, bottom right). More evidence is needed to address this question, which may help us have a deep insight into the mechanism of apoptosis regulation by the DANCR/ QK/MALAT1 axis.

Altogether, this study identified a Dox-regulated lncRNA DANCR and explored the suppressive function of DANCR on apoptosis. DANCR controlled expression of the RNA-binding protein QK to enhance the stability of MALAT1. Both MALAT1 and QK could efficiently mediate the anti-apoptotic function of DANCR. These data revealed new evidence for understanding the biological functions and the downstream regulatory network of DANCR, which may help develop novel therapies targeting DANCR in colorectal cancer.

## Materials and methods

### Cell culture

Human colorectal cancer cell lines HCT116, RKO, SW620, HT-29, and LoVo were provided by the Cell Bank, Type Culture Collection Committee, Chinese Academy of Sciences (Shanghai, China). All cell lines were authenticated by STR profiling and tested for mycoplasma contamination. HCT116 and HT-29 cells were maintained in DMEM (Hyclone, Logan, UT, USA) supplemented with 10% FBS (PAN-Biotech, Bavaria, Germany) and 1% penicillin–streptomycin (Hyclone, Logan, UT, USA). RKO, SW620, and LoVo cells were maintained in RPMI 1640 (Hyclone, Logan, UT, USA) supplemented with 10% FBS and 1% penicillin–streptomycin. Cells were cultured at 37 °C in a humidified atmosphere with 5% CO_2_.

### Plasmids

Human *DANCR* and *QK* were amplified by PCR and cloned into the CD513B lentiviral vector (SBI System Biosciences, Palo Alto, CA, USA) to generate CD513B–DANCR and CD513B–QK overexpression plasmids. MALAT1 full length cDNA and a Luciferase reporter plasmid with *MALAT1* promoter were obtained from Fenghui Biotechnology (Hunan, China). pMS2-GFP (#27121) and pSL-MS2-12X (#27119) were obtained from Addgene. The pPyCAGIP vector was a kind gift from Dr. Austin Smith. Sequences of primers, siRNAs, and DNA oligos for indicated applications are listed in Table S1.

### Cell transfection

For transient transfection, cells were seeded into six-well plate at 30% confluence. siRNA transfections were performed with Lipofectamine RNAiMAX (Invitrogen, CA, USA) at a concentration of 90 nM. Plasmid transfections were performed with MegaTran 1.0 transfection reagent (OriGene Technologies, Rockville, MD, USA) according to the manufacturer’s instructions.

### RNA isolation and qRT-PCR

Total RNA was extracted from tissues and cells with TRIzol reagent (Molecular Research Center, Cincinnati, OH, USA). RNA (1 μg) reverse transcription (RT) was performed using a RevertAid First Strand cDNA Synthesis Kit (Thermo Scientific, Vilnius, Lithuania) in accordance with the manufacturer’s instructions. Quantitative real-time polymerase chain reaction (qRT-PCR) was performed on Roche Light Cycler 480 detecting system using LightCycler 480 SYBR Green Master (Roche, Indianapolis, IN, USA). Primer sequences for indicated genes were listed in Table S1. Relative mRNA expression was calculated with the 2^−ΔΔCt^ method, and gene expression levels were normalized to GAPDH. cDNA arrays derived from human colon cancer tissues (#HCRT101, #HCRT102, and #HCRT104 purchased from OriGene Technologies, USA) were detected with the indicated primers according to the manufacturer’s instructions. The relative expression levels of DANCR in cancer tissues were calculated with methods described above and compared to normal tissues.

### Western blotting

Total protein was extracted from cells using RIPA lysis buffer (Millipore, Temecula, CA, USA). After quantification with a bicinchoninic acid (BCA) protein assay kit (Thermo Scientific, Rockford, IL, USA), protein was denatured at 95 °C for 10 min, then separated by 10% or 15% SDS–PAGE and transferred to 0.22 or 0.45 μm PVDF membranes (Millipore, Co. Cork IRL). Then the membranes were blocked in 5% nonfat milk and incubated with specific primary antibodies at 4 °C overnight, followed by incubation with secondary antibody conjugated with horseradish peroxidase (HRP) (1:5000, CST, MA, USA).

Antibodies against ACTIN (cat. no. 4970), CASPASE 3 (cat. no. 9662) and Cleaved CASPASE 3 (Asp175) (cat. no. 9661), CASPASE 7 (cat. no. 12827) and Cleaved CASPASE 7 (cat. no. 9491), PARP (cat. no. 9542) and Cleaved PARP (Asp214) (cat. no. 5625), Anti-mouse (cat. no. 7076) and anti-rabbit (cat. no. 7074) IgG HRP-linked antibodies were all purchased from Cell Signaling Technology. QK antibody (cat. no. ab126742) was purchased from Abcam.

### Cell proliferation assay

The Cell Counting Kit-8 (CCK-8) assay (Dojindo, KMJ, Japan) was used to measure cell proliferation. 1000 cells with indicated treatment were seeded into 96-well plate. CCK8 regent was added at 0, 24, 48, 72, 96 h, and incubated at 37 °C for 3 h in the dark before measuring the absorption at 450 nm with a Microplate Reader (Tecan, Australia).

### Cell cycle analysis

Cell cycle was analyzed using a Cell Cycle Detection Kit (Beyotime, Shanghai, China). Briefly, cells were harvested and fixed in 70% cold ethanol overnight at 4 °C. Fixed cells were washed twice in cold PBS and treated with RNase A for 1 h. Cells were then stained with propidium iodide (PI) for 30 min and immediately analyzed with a flow cytometer (Becton Dickinson, CA, USA).

### Soft agar assay

The six-well plates were coated on the bottom with a layer of complete DMEM medium (20% FBS, 2% penicillin–streptomycin) mixed with 1.2% low melting point agarose. 500–1000 cells were suspended in complete DMEM medium (20% FBS, 2% penicillin–streptomycin) with 0.6% low melting point agarose and plated on top of the wells. Two weeks later, the clones were stained with crystal violet and statistically analyzed.

### CASPASE 3/7 activity assay

CASPASE 3/7 activity was measured with a CASPASE 3/7 Activity Assay Kit (Cell Signaling Technology, MA, USA). Briefly, cells with different treatments were collected in lysis buffer. 100 μg total proteins were added into a black 96-well plate, mixed with substrate solution and incubated at 37 °C in the dark. During the assay, activated CASPASE 3/7 cleaved the fluorescent substrate (Ac-DEVD-AMC), generating highly fluorescent AMC that can be detected using a fluorescence reader with the excitation at 380 nm and the emission at 450 nm. The RFU were read with a fluorescence reader at 3 h post-incubation and analyzed.

### Annexin V/7-AAD flow cytometry assay

Cells with different treatments were collected and suspended in 100 μl binding buffer, containing 5 μl Annexin V-PE and 5 μl 7-AAD. Samples were then incubated for 30 min at room temperature in the dark, and the reaction was terminated with 400 μl binding buffer. The apoptotic cell population was detected by a flow cytometer (Becton Dickinson, San Diego, CA, USA).

### TdT-mediated dUTP nick-end labeling (TUNEL) assay

The TUNEL assay was performed with the In Situ Cell Death Detection Kit (Roche, Mannheim, Germany; cat. no. 11684795910). Cells were fixed with 4% paraformaldehyde for 1 h at room temperature and washed twice with phosphate buffered saline (PBS). The samples were permeated with 0.1% Triton X-100 in sodium citrate for 2 min on ice and rinsed twice in PBS. TUNEL reaction mixture was incubated with samples in a humidified atmosphere for 60 min at 37 °C in the dark. The nuclei were stained with DAPI. The apoptosis ratio was calculated by counting the TUNEL-positive cells. Imaging was performed with Leica microsystems (Leica, Germany).

### Xenograft tumor formation

Four weeks old male BALB/c-nude (nu/nu) mice were obtained from the Laboratory Animal Center of Sun Yat-Sen University and maintained in specific pathogen-free (SPF) environment. The procedure of animal experiments was approved by the Institutional Animal Care and Use Committee of Sun Yat-Sen University. 3 × 10^6^ shDANCR cells or control cells were injected subcutaneously into BALB/c-nude (nu/nu) mouse (*n* = 7 for each group). Tumor volumes were monitored by measuring the tumor diameter every week and were calculated using the formula (L*W^2^)/2. Four weeks later, the mice were sacrificed to harvest the tumors, which were weighed and subjected to IHC staining for Ki-67 and cleaved CASPASE 3.

### Luciferase reporter assay

To assess the function of DANCR on *MALAT1* promoter activity, HCT116 cells with control shRNA or shDANCR were transfected with a Luciferase reporter plasmid containing *MALAT1* promoter using MegaTran 1.0 (OriGene Technologies, Rockville, MD, USA). Cell lysate was harvested 48 h post-transfection, and Dual-Luciferase Reporter Assay System (Promega, Madison, WI, USA) was used to measure the relative Luciferase activity in accordance to the manufacturer’s instructions.

### RNA-sequencing analysis

Total RNA was extracted from cells using TRIzol reagent (Molecular Research Center, Cincinnati, OH, USA). RNA samples passed through quality control assessment were subjected to the second-generation high throughput sequencing (CapitalBio Technology). Gene expression levels were calculated by StringTie software as fragments per kilo bases per million fragments (FPKM). Differentially expressed genes were defined as fold change ≥ 1.5 and *p* < 0.05. Data of RNA sequencing can be accessed in NCBI GEO database (GSE145407).

### Fluorescence in situ hybridization (FISH)

The RNA fluorescence in situ hybridization was carried out using the ACDBio RNAscope multiplex fluorescence assay kit. RNA probes targeting DANCR (NR_024031.2) and MALAT1 (NR_002819.4) were synthesized by ACDBio Company. FISH was performed according to the manufacturer’s instructions. Briefly, cells cultured in an eight-well chamber (Thermo Fisher Scientific, USA) were fixed in 4% PFA for 15 min at room temperature, and then treated with hydrogen peroxide solution and Protease III for 10 min, respectively. MALAT1 probes (C2, Opal 520) were diluted 1:50 in DANCR probe (C1, Opal 570) and pipetted into each well. Probe hybridization took place at 40 °C for 2 h and cells were rinsed in 1× wash buffer. Cells were sequentially incubated in AMP1 for 30 min, AMP2 for 30 min and AMP3 for 15 min at 40 °C, rinsed with 1× wash buffer between each incubation, for signal amplification. Cells were also counterstained with DAPI to visualize nuclei. Samples were subsequently observed with Zeiss LSM 880 confocal microscope.

### Immunofluorescence staining

Cultured cells were fixed in 4% (v/v) paraformaldehyde and permeabilized with 0.2% Triton X-100 for 15 min The cells were first blocked with 10% normal goat serum for 1 h at room temperature, incubated with QK antibody (1:100 dilution) overnight at 4 °C, and then incubated with Alexa 555-labeled goat anti-rabbit secondary antibody (1:500 dilution) in the dark. Nuclei were visualized with DAPI (Fluka). Images were acquired using Zeiss 880 confocal microscope.

### RNA-binding protein immunoprecipitation

RIP assay was performed with Magna RIP RIP Kit (Millipore, Billerica, MA, USA). For RIP with QK antibody, 1 × 10^7^ HCT116 cells were rinsed twice with cold PBS and scraped off from plates and transferred to a centrifuge tube. Cells were collected by centrifugation at 1500 rpm at 4 °C and the supernatant was discarded. 100 μl RIP lysis buffer was added to the cell pellet and incubated the lysate on ice for 5 min and subsequently stored at −80 °C overnight. Thawed the lysate and centrifuged at 14,000 rpm for 10 min at 4 °C. Mixed 100 μl supernatant with 900 μl RIP immunoprecipitation buffer composed of 860 μl RIP wash buffer, 35 μl 0.5 M EDTA, and 5 μl RNase inhibitor. Incubated QK antibody (Abcam)-bound magnetic beads with cell lysate overnight at 4 °C with gentle rotation. After washing for five times, immunoprecipitated RNAs and proteins were purified for analysis. Western blotting was used to verify the specificity of QK antibody. qRT-PCR was applied to detect the RNAs enriched by QK.

For MS2-DANCR RIP, wild type HCT116 cells were first transfected with the following combinations of plasmids: (1) MS2+DANCR (BS) consisting of Flag-MS2-GFP/pPyCAGIP and DANCR-MS2 BS (12X)/pPyCAGIP; (2) MS2+DANCR consisting of Flag-MS2-GFP/pPyCAGIP and DANCR/pPyCAGIP; (3) DANCR (BS) consisting of DANCR-MS2 BS (12X)/pPyCAGIP alone. RIP assays were performed as described above with the anti-FLAG M2 Magnetic Beads (Sigma).

### Statistical analysis

All experiments shown were biologically replicated for more than five times. All results were shown as mean ± standard deviation from at least three independent experiments. Two-tailed Student’s *t*-test was performed for two groups, and one-way ANOVA for multi-group comparison. The xenograft tumor weight changes were compared using a repeated measure ANOVA. Statistical analyses were conducted using SPSS 22.0 software (SPSS, Inc., Chicago, IL, USA) and *p* < 0.05 was considered significant.

## Supplementary information

Supplemental Figure Legends

Supplemental Figure S1

Supplemental Figure S2

Supplemental Figure S3

Supplemental Figure S4

Supplemental Figure S5

Supplemental Figure S6

Supplemental Figure S7

Supplemental Figure S8

Supplemental Table S1

Supplemental Table S2

Supplemental Table S3
