# Supplementary material for: LncRNA DANCR represses Doxorubicin-induced apoptosis through stabilizing MALAT1 expression in colorectal cancer cells
Source: Cell Death Dis. 2021 Jan 6;12(1):24. doi: 10.1038/s41419-020-03318-8 (PMC7791116; doi:10.1038/s41419-020-03318-8)
Supplement: Supplementary file 1 — Supplemental Figure Legends [file 41419_2020_3318_MOESM1_ESM.docx]

**Supplemental figure legends**

**Figure S1. The functions of DANCR on the cell cycle progression, colony formation capacity, and the migration activity in colorectal cancer cells.**

(**A**) Relative gene expression of DANCR was detected with qRT-PCR in HCT116, SW620 and HT-29 cells with shDANCR. *, *p* < 0.05. (**B**) Relative expression level of DANCR was detected with qRT-PCR in HCT116 and SW620 cells overexpressing DANCR. *, *p* < 0.05. (**C**) Representative images of cell cycle progression analyzed in HCT116 (top) and SW620 (bottom) cells with DANCR depletion or DANCR overexpression by flow cytometry. PI, propidium iodide. (**D**) Representative images of colony forming assays using HCT116 and SW620 cells with shDANCR (left) and DANCR overexpression (right). (**E**) The expression levels of epithelial (E-CAD and CLDN-7) and mesenchymal (VIMENTIN) marker genes were detected by qPCR in HCT116 cells with DANCR depletion (top) or DANCR overexpression (bottom). *, *p* < 0.05; **, *p* < 0.01; ***, *p* < 0.001. (**F**) Representative images and statistical analysis of transwell assays showed that DANCR silencing had minor effects on cell migration in HCT116 and SW620 cells.

**Figure S2. DANCR suppressed apoptosis in HT-29 cells.**

(**A**) Western blot showed increased expression of total and cleaved PARP, CASPASE 7, and CASPASE 3 in HT-29 cells upon treatment with 200nM Doxorubicin for 24h.

(**B**) Flow Cytometry assays showed increased apoptotic cell numbers upon DANCR silencing in HT-29 cells treated with DMSO or 200nM Doxorubicin for 24h. Representative images and statistical analysis of the percentage of apoptotic cells were shown. *, *p* < 0.05.

**Figure S3. The transcription of MALAT1 was unaffected by DANCR.**

The activity of *MALAT1* gene promoter in HCT116 cells with shDANCR or control vector was evaluated by Luciferase reporter assay.

**Figure S4. The efficiency of MALAT1 knockdown and overexpression in HCT116 cells.**

Relative gene expression of MALAT1 was detected with qRT-PCR in HCT116 cells with MALAT1 ASO (**A**) or cells overexpressing MALAT1 (**B**). *, *p* < 0.05.

**Figure S5. The subcellular localization of DANCR and MALAT1.**

Fluorescence in situ hybridization identified the subcellular localization of DANCR and MALAT1 in HT-29 and HCT116 cell lines. Red: DANCR, Green: MALAT1, Blue: DAPI. Arrow: Co-localization of DANCR and MALAT1. Bar: 10μm.

**Figure S6. The enrichment of QK mRNA, MALAT1 and U1 from MS2-DANCR RIP assays.**

Relative gene enrichment (% input) of DANCR, QK mRNA, MALAT1 and U1 from MS2-DANCR RIP assays was detected by qRT-PCR. QK mRNA, MALAT1 and U1 did not show consistent enrichment in the MS2+DANCR (BS) group compared to the two negative controls MS2+DANCR and DANCR (BS). *, *p* < 0.05.

**Figure S7. The efficiency of QK knockdown and overexpression in HCT116 and SW620 cells.**

Relative gene expression of QK was detected by Western Blotting and qRT-PCR in HCT116 (**A**) and SW620 (**B**) cells with siQK or a QK overexpression vector. *, *p* < 0.05.

**Figure S8. The inputs of MALAT1, QK mRNA, and DANCR in the RIP assay with QK antibody.**

The expression levels of MALAT1, QK mRNA, and DANCR from the cell lysate inputs of the QK RIP assay were detected by qRT-PCR. *, *p* < 0.05.
