# Supplementary figures and images for: LncRNA DANCR represses Doxorubicin-induced apoptosis through stabilizing MALAT1 expression in colorectal cancer cells
Source: Cell Death Dis. 2021 Jan 6;12(1):24. doi: 10.1038/s41419-020-03318-8 (PMC7791116; doi:10.1038/s41419-020-03318-8)

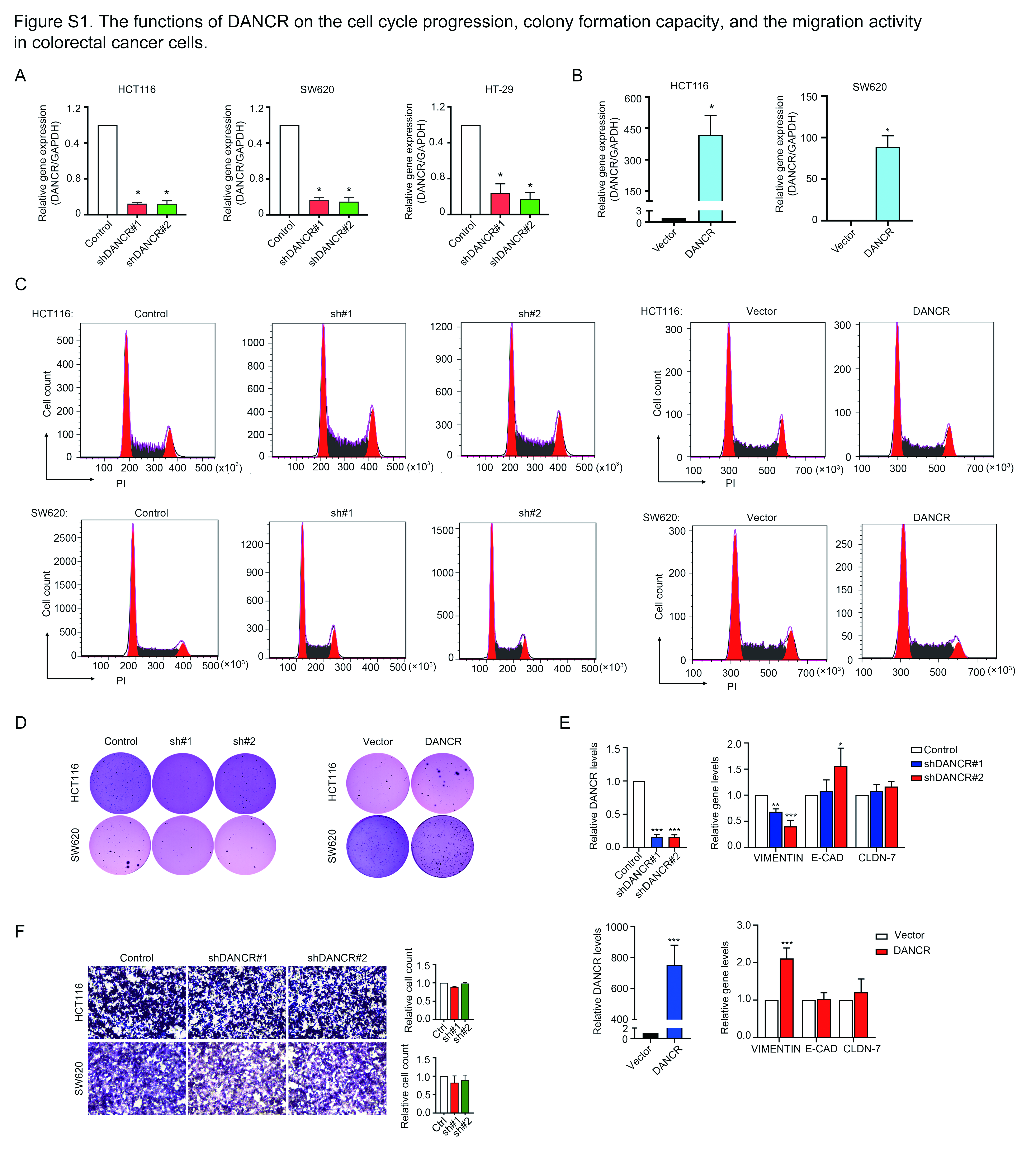

Supplement: Supplementary file 2 — Supplemental Figure S1 [file 41419_2020_3318_MOESM2_ESM.tif]

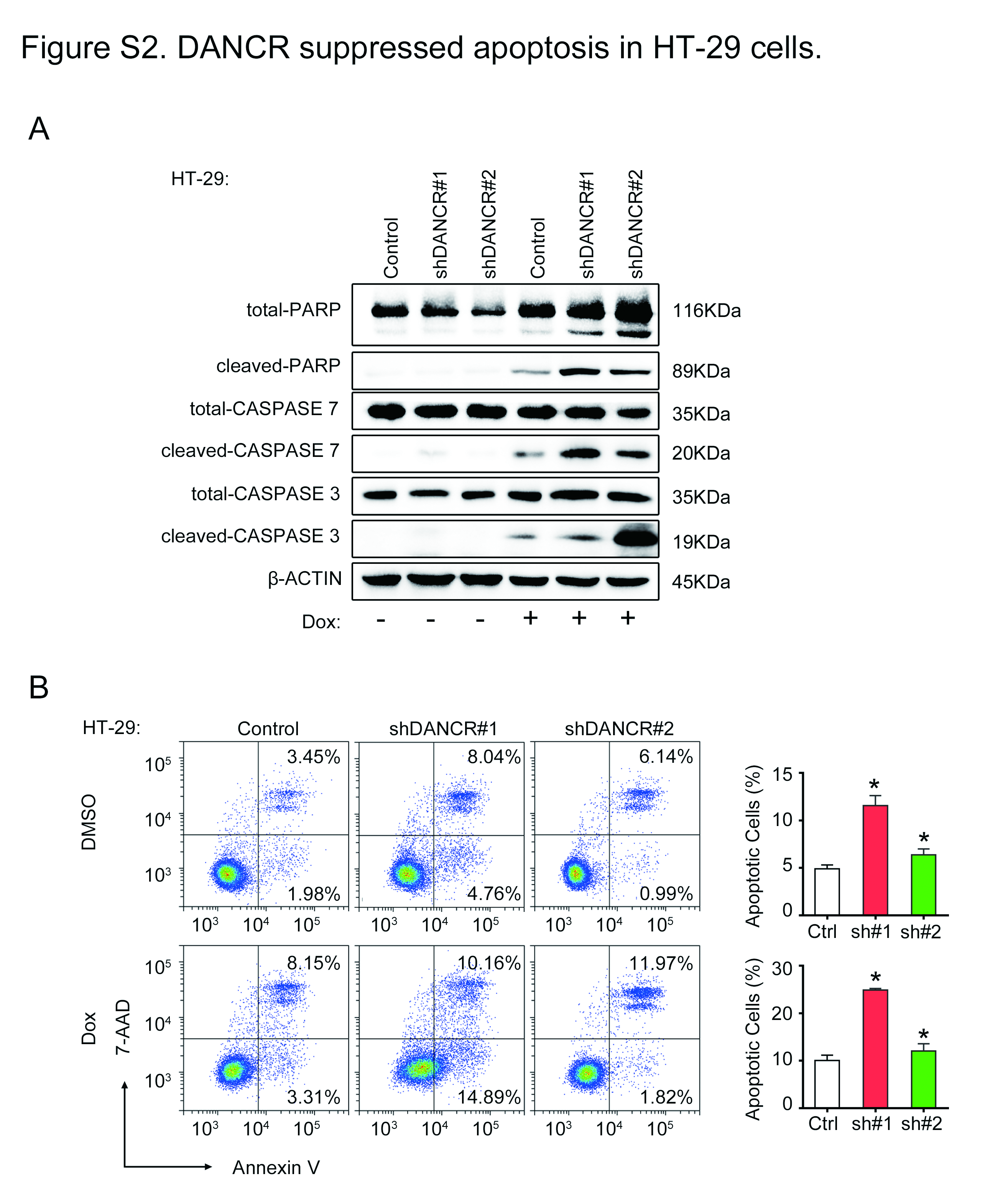

Supplement: Supplementary file 3 — Supplemental Figure S2 [file 41419_2020_3318_MOESM3_ESM.tif]

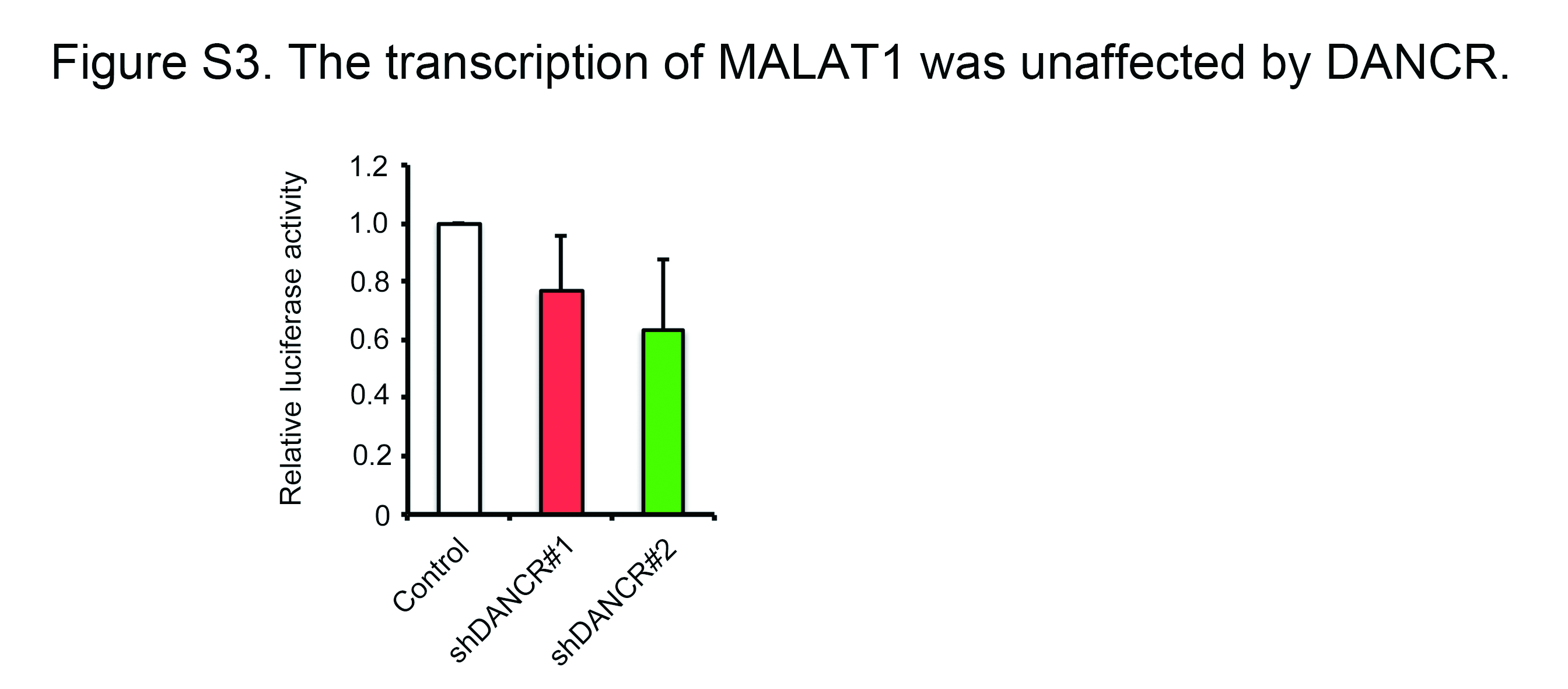

Supplement: Supplementary file 4 — Supplemental Figure S3 [file 41419_2020_3318_MOESM4_ESM.tif]

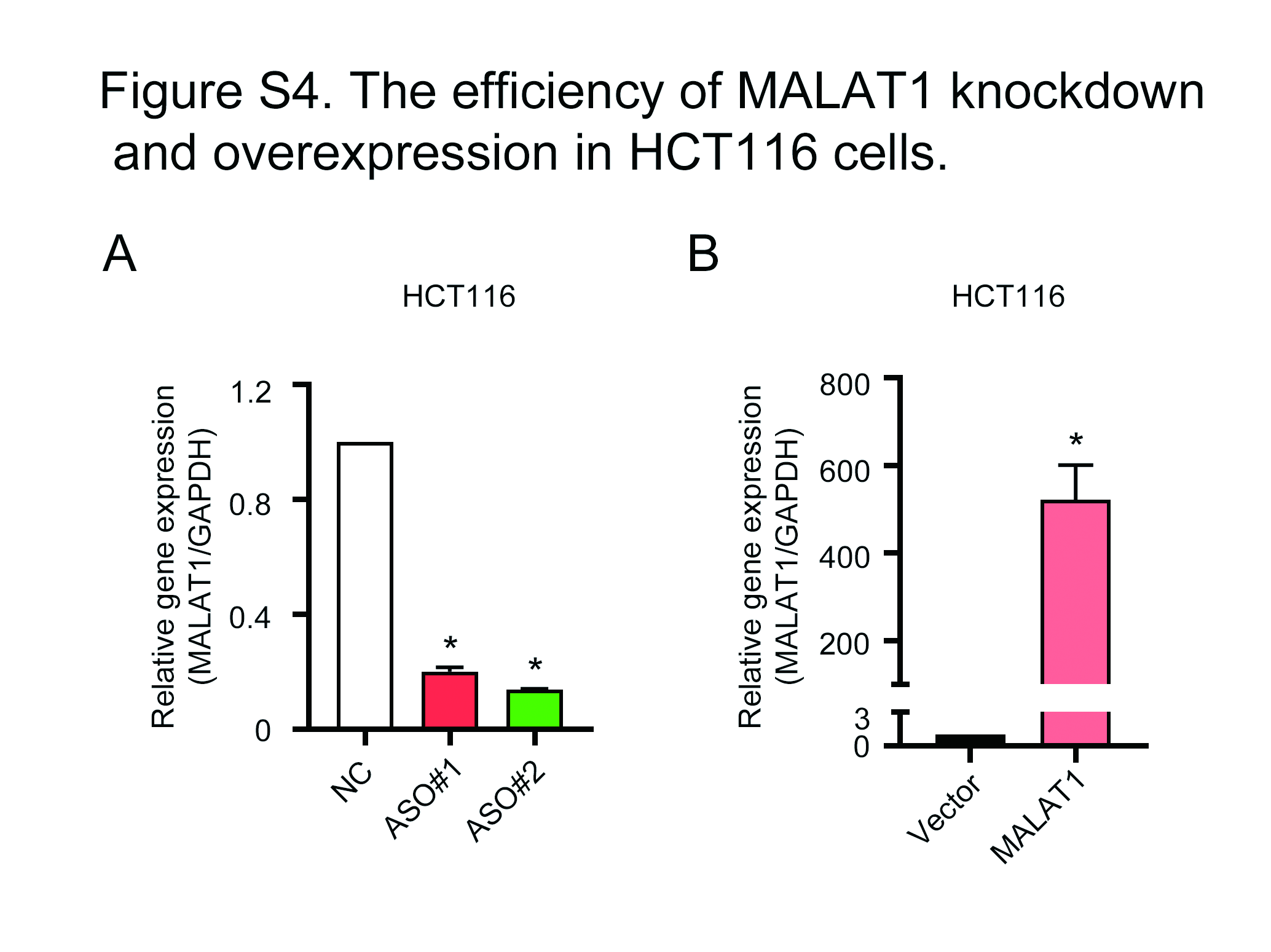

Supplement: Supplementary file 5 — Supplemental Figure S4 [file 41419_2020_3318_MOESM5_ESM.tif]

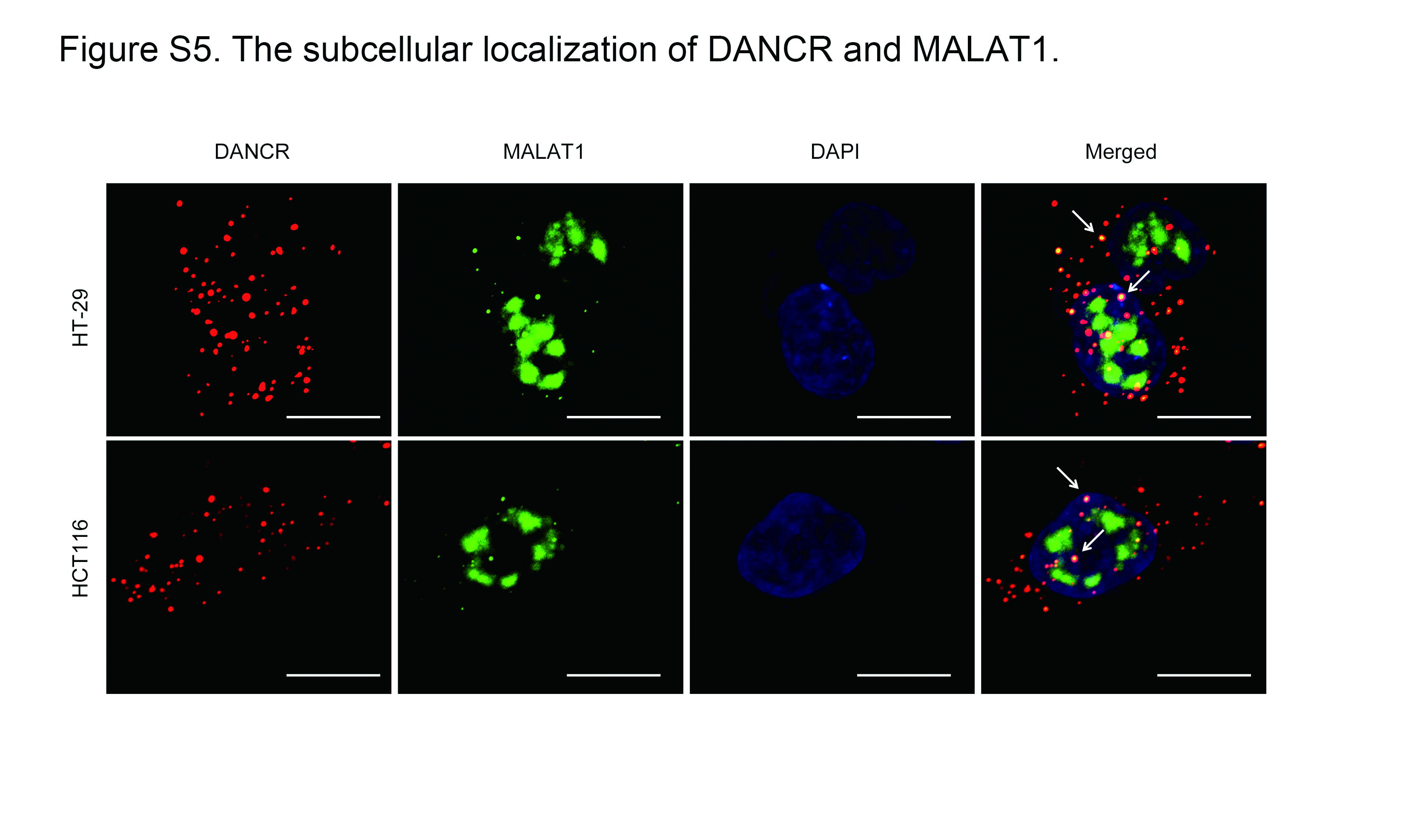

Supplement: Supplementary file 6 — Supplemental Figure S5 [file 41419_2020_3318_MOESM6_ESM.tif]

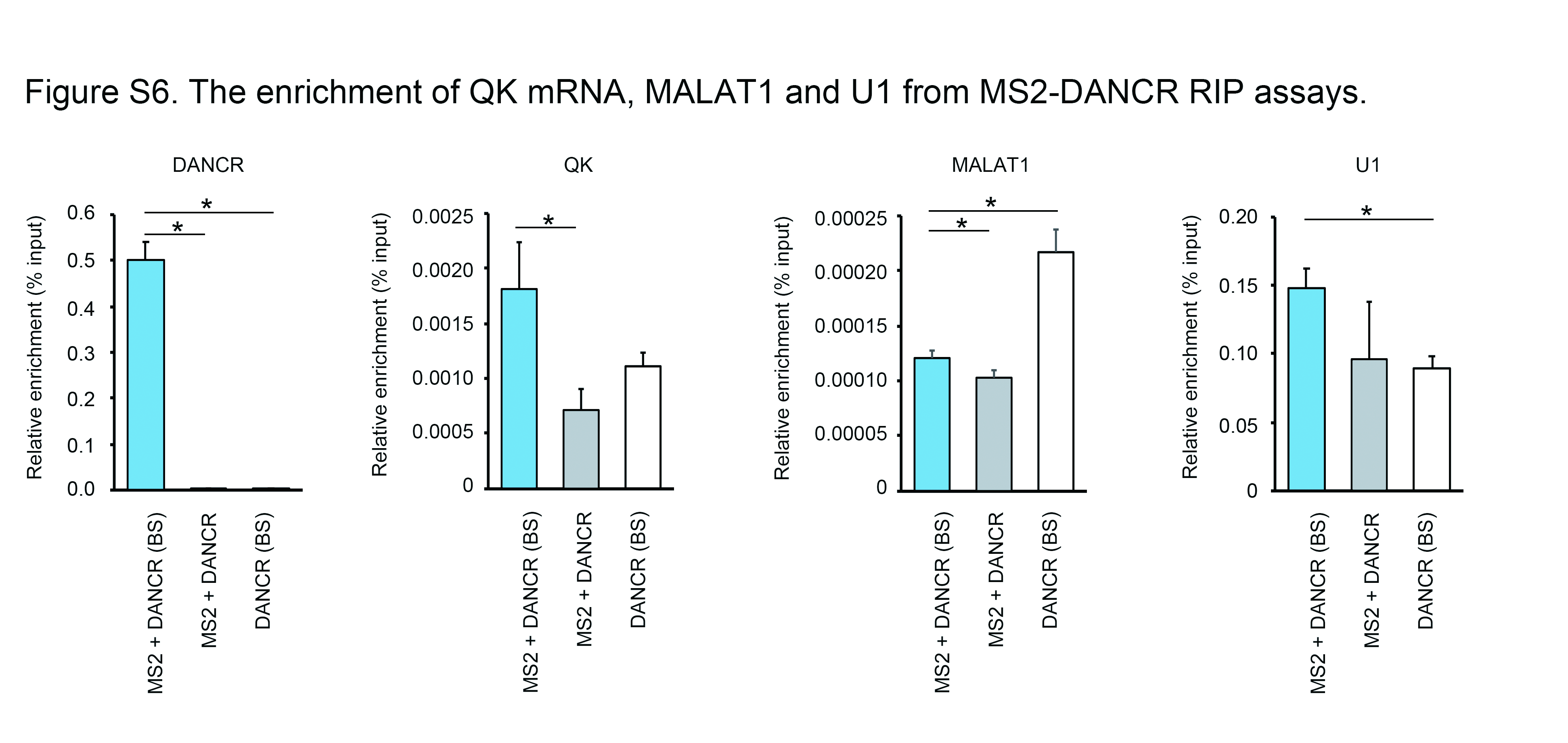

Supplement: Supplementary file 7 — Supplemental Figure S6 [file 41419_2020_3318_MOESM7_ESM.tif]

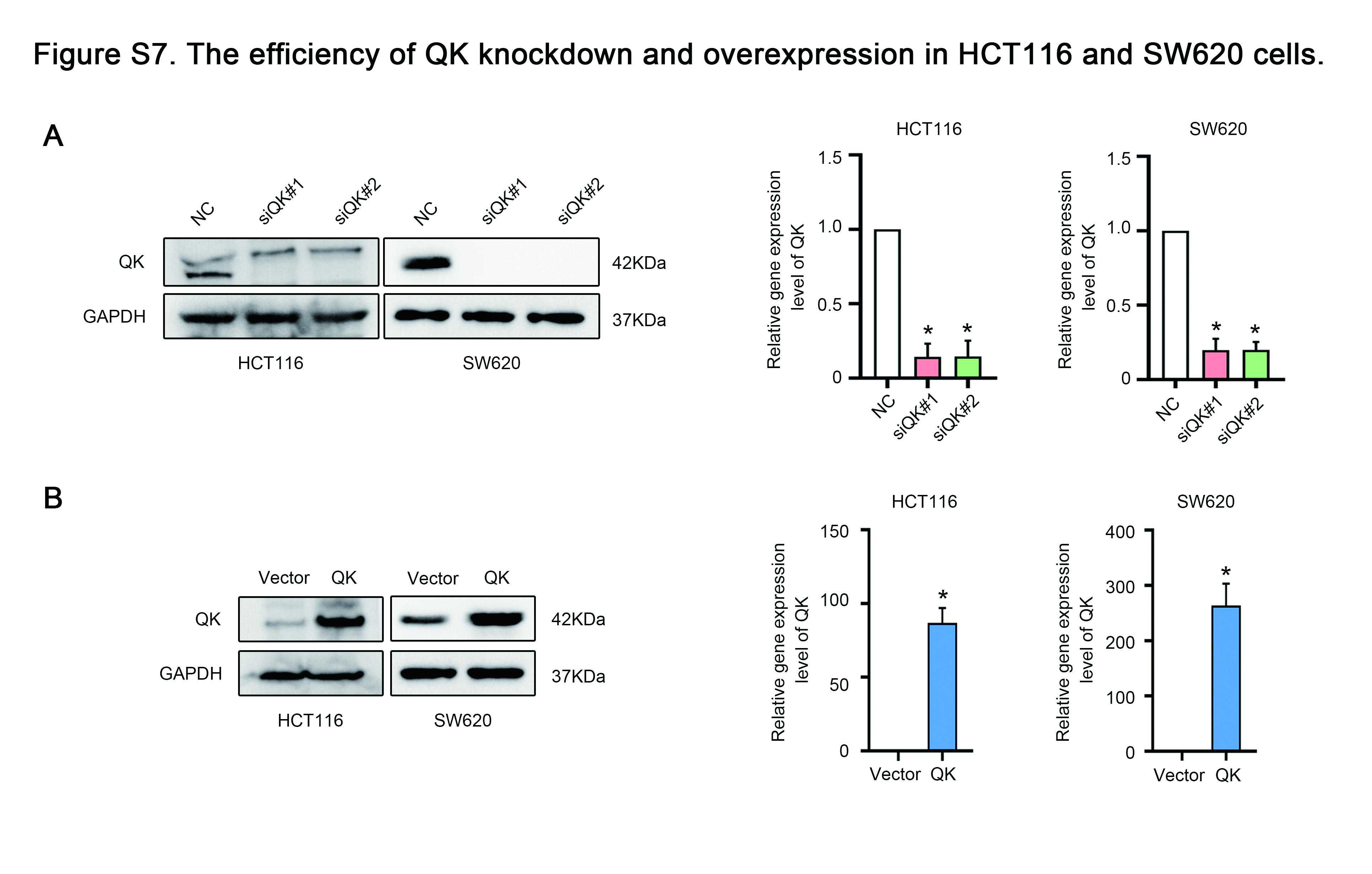

Supplement: Supplementary file 8 — Supplemental Figure S7 [file 41419_2020_3318_MOESM8_ESM.tif]

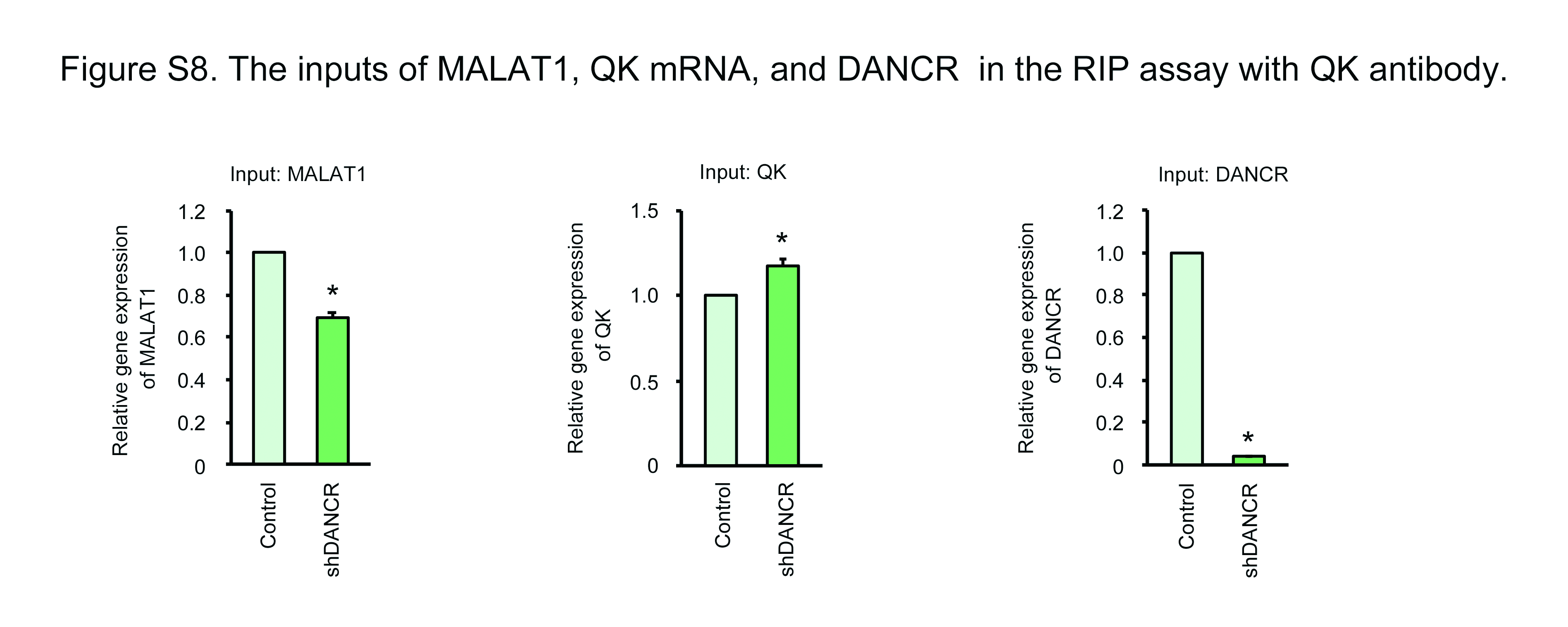

Supplement: Supplementary file 9 — Supplemental Figure S8 [file 41419_2020_3318_MOESM9_ESM.tif]
